# Current landscape of paroxysmal nocturnal hemoglobinuria in the era of complement inhibitors and regulators

**DOI:** 10.1177/20406207241307500

**Published:** 2024-12-23

**Authors:** Julia J. Shi, Yusuf M. Ozcan, Carlos I. Ayala Santos, Hetalkumari Patel, Jamile Shammo, Taha Bat

**Affiliations:** Division of Hematology and Oncology, Department of Internal Medicine, University of Texas Southwestern Medical Center, Dallas, TX, USA; University of Texas at Dallas, Richardson, TX, USA; Division of Hematology and Oncology, Department of Internal Medicine, University of Texas Southwestern Medical Center, Dallas, TX, USA; Department of Pharmacology, University of Texas Southwestern Medical Center, Dallas, TX, USA; Feinberg School of Medicine, Northwestern University, Chicago, IL, USA; Division of Hematology and Oncology, Department of Internal Medicine, University of Texas Southwestern Medical Center, 5323 Harry Hines Blvd., Dallas, TX 75390-9255, USA

**Keywords:** complement cascade, complement inhibitors, PNH

## Abstract

Paroxysmal nocturnal hemoglobinuria (PNH) is a rare blood disorder which is caused by mutations in phosphatidylinositol glycan class A leading to hemolysis of red blood cells via complement inhibition. The first treatment for PNH, eculizumab, was FDA approved in 2007. Since then, many new treatment options for PNH have arisen. This critical review will examine all medications available for PNH on the US market, highlight several major medications in development, and discuss the risks and treatment considerations associated with each option. It is not intended to address PNH clonal dynamics, disease presentation, or discussions on when to initiate treatment.

## Plain language summary

Current treatment options for paroxysmal nocturnal hematuria and prices

Treatment options for paroxysmal nocturnal hematuria have changed significantly since 2007, when the first medication for this disease was approved in the United States. This review begins by explaining the biochemical pathways that explain the pathophysiology of paroxysmal nocturnal hemoglobinuria. Then, various effects of the disease and treatments including breakthrogh intravascular hemolysis and extravascular hemolysis are explained. Finally, each treatment option for paroxysmal nocturnal hemoglobinuria is discussed and compared in terms of effectiveness, price, and how often the patient needs to take the medication.

## Introduction

Paroxysmal nocturnal hemoglobinuria (PNH) is a rare pan-bone marrow disorder caused by complement mediated hemolysis of PNH red blood cells (RBCs) and increased thrombus formation, with one study in the United Kingdom estimating its prevalence at roughly 4 per 100,000 people and incidence of 0.57 per 100,000 person-years.^1^ PNH is caused by mutations in the phosphatidylinositol glycan class A (*PIG-A*) gene, responsible for coding the glycophosphatidylinositol (GPI) anchor proteins, which lead to a deficiency or complete absence of the hematopoietic surface receptors CD55 and CD59.^2^ An absence of these complement regulatory proteins renders PNH RBCs susceptible to membrane attack complex-related destruction by the complement system, therefore causing premature death by lysis. This carries significant risk of early mortality due to anemia and thromboembolic events: at 10 years, survival among PNH patients was 68.4% (vs 85.8% in general population).^3^ Thrombus formation in the major veins and cerebrum leads to end organ failure and even death. Thrombus formation in the portal triad is particularly common, leading patients with PNH to sometimes present with hepatomegaly as well as jaundice.

Over the past two decades, treatment for PNH has undergone a revolutionary transformation following key discoveries such as the identification of the *PIG-A, X*, and *T* gene mutation and the clonal nature of the disease. Central to this transformation are medications which modify the complement cascade, from complement inhibitors to complement regulators.

### PNH classification and clinical significance of PNH clone

There are three classifications of PNH according to the International PNH Interest Group. Patients with “classic PNH” display clinical symptoms such as hemolysis, abdominal pain, and dysphagia, alongside a substantial PNH clone size (>50%).^4^ Patients with “PNH in the context of another bone marrow disorder” do not have a PNH disease and instead have an accompanying PNH clone, thus usually present with lower PNH clone size (20%–30%) and primarily exhibit symptoms of the underlying bone marrow disorder. For example, in conditions like acquired aplastic anemia (AA), PNH clones can evade immune attacks and expand, leading to larger populations. PNH clones very seldom can also arise in myelodysplastic syndrome and myeloproliferative neoplasms, primarily associated with immune-mediated bone marrow failure. Clinical presentations of PNH vary, with classical PNH featuring hemolysis and large clone sizes, while most bone marrow failure patients with PNH are asymptomatic and have smaller clones. Finally, patients with “subclinical PNH” lack symptoms, yet still have detectable and substantial PNH clone size (<20%). In healthy individuals, although polyclonal PNH cells exist in very small populations (0.001%–0.005%), they do not significantly expand without pathology.^5,6^ More recently, a potential distinct presentation of PNH has been introduced known as ahemolytic PNH.^7^

### Complement cascade overview and relation to PNH disease

The complement system, a vital component of the innate immune response comprising over 50 distinct plasma and serum proteins, orchestrates the opsonization of pathogens and the initiation of inflammatory responses to combat infections. Activation of the complement cascade occurs through three distinct pathways (classical, lectin, and alternative), each triggered by different molecules, but all converging at the C3 point to generate a uniform set of effector molecules. The classical pathway is activated by antibodies or direct binding of complement component C1q to pathogen surfaces, while the lectin pathway is triggered by mannan-binding lectin, a normal serum constituent that binds certain encapsulated bacteria. In contrast, the alternative pathway, which remains continuously active at a low level, is directly triggered on pathogen surfaces. Upon activation, a complement protein is cleaved into its active form, initiating a cascade of cleavage events that convert other proteins into their active forms. This cascade culminates in proteolytic, inflammatory, and lytic processes, effectively combating invading pathogens.

The complement cascade serves a dual role of immune surveillance and host defense, tagging pathogens for removal, inducing inflammatory responses, and recruiting and activating phagocytes to sites of complement activation. Regulation of complement activity is crucial to restrict the process to invading pathogens and prevent damage to healthy host tissues. Regulatory proteins and inhibitors play a crucial role in ensuring that potent effectors are tightly controlled. Additionally, RBCs are potentially sensitive to lysis compared to platelets and granulocytes. CD59 and CD55, as cell-surface proteins, are part of the host’s self-recognition mechanism, protecting RBCs from destruction if the complement system is activated during infection. In PNH RBCs, lack of CD55 and CD59 leads to early destruction by complement cascade activation.

### PNH clonal expansion or regression

The dynamics of PNH clones present a paradox that remains unresolved. Approximately 50% of patients with AA have PNH mutant cells at diagnosis, and a significant proportion, up to 40%, progress to develop overt hemolytic PNH.^8^ The expansion of PNH clones is associated with severe AA, the initial size of the granulocyte clone, and exposure to ATG, a treatment for AA. However, spontaneous remissions of PNH clones are rare and pose an intriguing puzzle. One speculation is that PIGA clones may be outcompeted by CHIP-like lesions in myeloid genes, potentially offering a fitness advantage. Alternatively, the reduction or disappearance of a PNH clone could result from the restoration of normal hematopoiesis, a phenomenon often seen in AA patients who respond well to immunosuppressive treatment.^9^

### Extravascular hemolysis definition

Over 50% of anti-C5 treated patients might have C3d deposition on their red cell shown by direct antiglobulin (DAT) testing and 10%–30% will continue to experience anemia and require transfusions mainly because of extravascular hemolysis (EVH). EVH is thought to occur because of C3 deposition on surviving, yet defective, RBCs, making them susceptible to removal by the liver or spleen. This process does not release lactate dehydrogenase (LDH) into the blood as much as intravascular hemolysis, making it challenging to assess EVH, though some patients do show hyperbilirubinemia. There is not yet a universal definition for clinically significant EVH (csEVH), and its presentation can be a combination of symptomatic anemia (hemoglobin (Hb) levels ⩽ 9.5 g/dL) with absolute reticulocyte count ⩾120 × 10^9^/L, and with or without blood transfusion.^10^

### Breakthrough hemolysis definition

Breakthrough hemolysis (BTH) is commonly described as the sudden return of symptoms related to intravascular hemolysis, such as hemoglobinuria, accompanied by a significant rise in LDH levels and a sharp drop in hemoglobin levels with or without major adverse vascular event. This phenomenon can occur due to inadequate inhibition of C5, which can be caused by factors affecting drug levels or drug effects, such as intense complement activation during infection, inflammation exceeding the C5 blockade, or any complement activating condition.^11,12^ In cases where BTH is due to pharmacodynamic reasons, a high concentration of C3b on RBC surfaces can create highly effective C5 convertases, which can cleave C5 despite the presence of an inhibitor.^13^ Additionally, the high concentration of C3b may cause a change in the shape of C5, activating it without cleaving it.^14^ BTH under these circumstances is typically unprecedented but transient, likely because the inhibitor regains control of C5 as the excess complement activation subsides. Inadequate dosing of C5 inhibitors can lead to incomplete inhibition of intravascular hemolysis. This is often observed as BTH toward the end of the dosing interval, accompanied by elevated levels of LDH.^11^

### Current treatment landscape for PNH

#### Eculizumab

**Figure 1.**
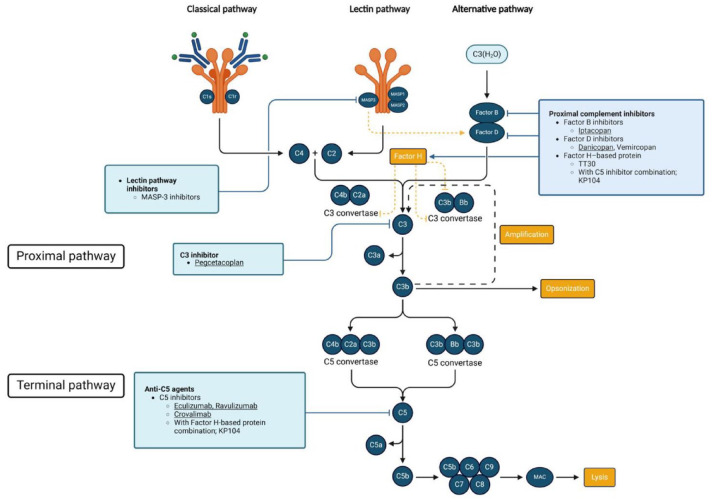
All available complement cascade modifiers are listed. FDA-approved medications are underlined. Figure created using BioRender.

The first medication for PNH, eculizumab, was FDA approved in 2007 and remained a mainstay of PNH treatment until longer acting Ravulizumab approval (Figure 1). Eculizumab is a humanized monoclonal antibody which acts as a terminal complement inhibitor. It binds to C5, preventing C5 cleavage and thus inhibiting the formation of the terminal complement complex, C5b-9.^15^ In SHEPHERD, a 52-week phase III open-label clinical trial of eculizumab, regular intravenous infusions resulted in 87% reduction in hemolysis, 51% of patients achieving transfusion independence.^16^ This was achieved through protection of PNH RBCs, with median PNH type III RBCs rising from 33.5% at baseline to 55.7% at week 52.^16^ Thus, eculizumab was shown to be highly effective at reducing hemolysis of PNH RBCs and thus ameliorating the anemia experienced by patients with PNH. In addition, the TRIUMPH study, a phase III double-blinded placebo-controlled trial, demonstrated that eculizumab treatment led to a remarkable reduction in intravascular hemolysis as well as thrombosis. In addition, clinical improvements in fatigue were reported.^17^

While eculizumab achieved good overall control of hemolysis, there were still a few cases of thrombotic events and infection concerns. Two patients with a history of thrombosis experienced a thrombotic event during SHEPHERD, though these events were considered by the study investigators to be unrelated to treatment.^16^ In addition, 89 patients experienced at least one infection, of which 8.3% were considered related.^16^ It has been proposed that inhibition of terminal complement through eculizumab results in higher risk of infection by microorganisms normally targeted by terminal complement such as *Neisseria meningitidis* and *Neisseria gonorrhoeae.*^18^ Thus, for patients planning to initiate eculizumab treatment, it is critical to vaccinate against *Neisseria* species at least 2 weeks before treatment. In addition, 2.1% of patients receiving eculizumab developed anti-eculizumab antibodies, although these were in low titers and ultimately did not impact efficacy of the drug. Thus, the anti-eculizumab antibodies are likely of limited clinical significance.^16^ Eight patients in the SHEPHERD trial demonstrated a return of terminal complement activity and hemolysis—BTH—during the last 1 or 2 days of the 14-day dosing interval.^16^ This was resolved by adjusting the dosing interval to 12 days instead of 14.^16^ This suggests that the BTH was due to inadequate eculizumab dose and thus inadequate control of complement.

BTH is a relatively new concept in PNH treatment and is typically attributed to insufficient drug dose or overly active complement. In the case of eculizumab, it has been suggested that BTH occurs in both cases: because of insufficient dose requiring more frequent infusions, or because of strong complement activation which overrides the terminal pathway inhibition by the anti-C5 antibody eculizumab. The strong complement activation leads to higher C3b density, which induces a conformation change limiting the ability of eculizumab to block terminal complement and causing BTH.^19,20^ The idea that the patient experiences BTH because of insufficient eculizumab to control complement is supported by the fact BTH rates increase at dosing of 17 days or more.^21^

Moreover, the concept of controlling BTH with adequate dose of eculizumab is further reinforced by the fact all patients with free eculizumab residual levels of 150 µg/L or more never experienced a breakthrough.^22^ However, given the cost of eculizumab is over $600,000 per year, simply increasing the dose for all patients to prevent BTH is not a realistic plan. Instead, there has been a rising interest in monitoring complement blockade with eculizumab to ensure adequate efficacy and only escalating the dosage for patients in need. CH50 has arisen as a potential marker of eculizumab efficacy.^22^

BTH is especially common in patients with PNH who are pregnant, which is a scenario which increases complement activation. While eculizumab provides benefit in pregnancy—as evidenced by high rate of fetal survival and low rate of maternal complications—it also results in BTH rates of 35.7%–53.7%.^23,24^ When considering all the patients with PNH, approximately 11%–27% may experience BTH on approved doses of eculizumab.^25^

Thus, BTH in eculizumab is associated with lack of inhibition of C5 (insufficient dose) or cases of strong complement activation (pregnancy, infection, etc.). Eculizumab is the only agent that has been used in pregnant patients.^24^ These cases can be resolved by increasing the dosage, and patients ultimately do well on this drug.

Eculizumab also sees EVH due to targeting only C5. This is due to the accumulation of C3b on PNH RBCs which can occur in up to 50% of patients on C5 inhibitor, and can lead to some patients (up to 25%) developing csEVH with transfusion dependency.^26^

#### Ravulizumab

Ravulizumab is a long-acting C5 inhibitor which was FDA approved in 2018. It is a modified form of eculizumab which also binds to the complement C5 protein and is thus an anti-C5 therapy. However, while eculizumab is degraded in the endosome, ravulizumab is recycled by the FcRn-receptor and can bind to a second molecule of C5, meaning patients are able to receive fewer infusions/go longer between doses.^27^

In the 301 and 302 studies, two phase III studies showing noninferiority to eculizumab, patients treated with ravulizumab had equal control of hemolysis and anemia. They also experienced less BTH than those treated on eculizumab (301 (complement inhibitor−naïve patients), 5 vs 13, 302 (patients stabilized on eculizumab at baseline), 0 vs 5).^11,25,28^ In contrast with eculizumab, none of the BTH events in ravulizumab were associated with lack of adequate C5 inhibition versus 50% of eculizumab BTH cases were associated with that. Instead, in study 301, 80% of ravulizumab cases were associated with complement-amplifying conditions, specifically infection, (last patient had no temporal data) versus just 40.0% of eculizumab breakthrough events.^11^ In the 302 study, no ravulizumab patients experienced BTH versus 42.9% of eculizumab BTH associated with overly active complement. One patient left the study due to lack of efficacy of eculizumab.^25,28^

A large part of this difference is likely attributable to the fact ravulizumab has a 4x longer half-life than eculizumab.^29^ This means that ravulizumab can provide sustained control of C5, while eculizumab loses control of C5 as it completes more and more half-lives. Thus, it follows that ravulizumab is more associated with BTH in cases of overly active complement (C3) rather than lack of adequate inhibition of C5. Similar to eculizumab, ravulizumab carries infection risk by microorganisms typically targeted by terminal complement such as *N. meningitidis* and *N. gonorrhoeae.*^18,^30^^ These infections are associated with most cases of ravulizumab breakthrough, thus similar to eculizumab, patients planning to begin ravulizumab treatment should be vaccinated against *Neisseria* species at least 2 weeks before initiating treatment. There is currently no data on the use of Ravulizumab during pregnancy, raising concerns about its FcRn-mediated recycling mechanism and potential implications for fetal exposure. The survival of patients with eculizumab and ravulizumab was significantly lower than age- and sex-matched controls (P = .001), but after excluding 4 deaths due to thromboses and patients requiring treatment for bone marrow failure (n = 389), their survival was not significantly different from age- and sex-matched controls.^26^

It has been suggested that infections associated with BTH cause proximal complement activation which thus causes BTH.^31^ Thus, even though ravulizumab seems to have eliminated the breakthrough hemolytic events related to suboptimal free C5 inhibition, a nonnegligible fraction of patients still experiences various degrees of EVH (up to 20%) because of C3 fragment opsonization with clinically signficant transfusion dependency.^26^ To overcome this, further proximal complement inhibitor medications are being developed. However, similar to eculizumab, ravulizumab targets C5 and does not address residual anemia caused by EVH.

#### Pegcetacoplan

Pegcetacoplan is a PEGylated penta-decapeptide which inhibits proximal complement through C3. It is especially indicated for patients on eculizumab or ravulizumab experiencing EVH. Initial results of the PEGASUS trial, a phase III trial, showed that pegcetacoplan outperformed eculizumab in terms of hemoglobin improvement with a difference of 3.84 g/dL versus 9 g/L.^16,32^ Moreover, 85% of patients achieved transfusion independence. However, 10% of pegcetacoplan patients and 23% of eculizumab patients experienced BTH.^32^ In fact, 3 of 41 patients discontinued pegcetacoplan and transitioned to eculizumab due to BTH. This hemolysis was associated with a rapid increase in LDH level (intravascular hemolysis), though no direct cause was determined.^32^ Two of the three patients who had BTH on pegcetacoplan had lower than average value at steady state of pegcetacoplan prior to breakthrough. This may indicate an insufficient dose effect.^32^ A more recent study on the real-world use of pegcetacoplan showed 27% of patients experienced BTH.^33^ These events were able to be managed by prompt infusion of pegcetacoplan for 3 days (increasing dose).^33^ Thus, while pegcetacoplan allowed more patients to achieve increased hemoglobin levels, and thus freedom from anemia symptoms, there remains the problem of BTH.

The PEGASUS trial also showed that 29% of patients in the pegcetacoplan group and 26% in the eculizumab group had an infection.^32^ There are inherent problems with suppressing proximal complement, though the risk seems similar to that of terminal complement inhibitors, and no specific microorganisms were identified.

#### Iptacopan

Iptacopan is a Factor B inhibitor which acts on the alternative pathway in the complement cascade. Factor B is an upstream molecule to C3. As another proximal complement inhibitor, it is thought that iptacopan also addresses EVH. In fact, iptacopan has been used in addition to eculizumab in patients who continue to have intravascular hemolysis or C3 mediated EVH with eculizumab.^34^ It is also available as a monotherapy, with the APPLY-PNH and APPOINT-PNH phase III trials showing that patients had increases in hemoglobin level without transfusion.^12,35^ The studies also showed reduced fatigue and reduced reticulocyte and bilirubin levels. There is increased interest in iptacopan because it is an oral therapy, allowing for much greater flexibility for patients than an intravenous infusion.

The use of iptacopan with anti-C5 has been successful, with complete control of residual hemolysis.^34^ As a monotherapy, most of the recent literature shows 10 breakthrough events that occurred in 136 patients (7%), of which 8 were associated with complement amplification events such as COVID-19, infections or pregnancy.^12^ While these events were generally not severe and managed successfully with RBC transfusions and—in one case—eculizumab, iptacopan inherently carries a higher risk of severe BTH because the drug achieves such high levels of PNH RBCs (90%+).^34^^[Bibr bibr11-20406207241307500]^

In APPLY-PNH, assessing iptacopan monotherapy in a 24-week period, 51/60 patients had increased hemoglobin levels of at least 2 g per deciliter.^35^ In APPOINT-PNH, 31/33 patients had an increase of at least 2 g/dL of hemoglobin. Iptacopan also achieved control of extravascular and intravascular hemolysis markers.^35^ Thus, iptacopan achieved better control of EVH and thus anemia than anti-C5 therapy, though the risk of severe BTH remains.

As a complement inhibitor, iptacopan also carries infection risks. In the study of 136 patients, 5 patients experienced 13 infections.^34^ However, these all resolved without complication, showing noninferiority to infection risk with previous therapies.

#### Crovalimab

A new anti-C5 antibody which has enhanced recycling, crovalimab, was FDA-approved and has the potential to require even smaller and less-frequent doses than ravulizumab or eculizumab.^36^ In clinical trial COMMODORE III, crovalimab was able to be dosed via subcutaneous route once every 4 weeks, and self-administered by patients without medical supervision after adequate in-clinic training. Thus, crovalimab represents another convenient C5 inhibitor option for patients with PNH. In addition, none of the patients in the trial experienced meningococcal infections, though common adverse events such as viral infections and infusion site reaction were consistent.^37^

A potential drawback of crovalimab is the immune complex rash that develops on the skin after switching from eculizumab or ravulizumab therapy. However, latest studies show that all transient immune complex reactions are resolved with continued treatment.^38^

#### Danicopan

Danicopan is the first factor D inhibitor, which acts on the alternative complement pathway. ALPHA, an ongoing phase III trial of 73 patients evaluating danicopan as an add-on to anti-C5 therapies, has shown greater increases in hemoglobin versus anti-C5 alone with no new safety concerns.^39^ Thus, danicopan add-on therapy is suggested to improve hemoglobin concentration for patients with PNH and with clinically significant EVH. In addition, as an oral therapy, danicopan is much easier to administer compared with other medications on the market for PNH (Table 1).

**Table 1.** FDA-approved PNH medications.

Medication	FDA-Approval Date	PNH indication	Mechanism of action	Recommended vaccination
Eculizumab (Soliris™)	March 16, 2007	Adult patients with PNH	Humanized mAb against complement C5	Vaccinate against *N. meningitides* ⩾2 weeks before initiating treatment
Ravulizumab (Ultomiris™) (IV or SQ)	December 21, 2018	Adult and pediatric patients ⩾1 month of age with PNH		Vaccinate against *N. meningitides* ⩾2 weeks before initiating treatment
Pegcetacoplan (Empaveli™)	May 14, 2021	Adult patients with PNH	Binds C3 and activates fragment C3b	Vaccinate against *N. meningitides, Streptococcus pneumonia*, and *Haemophilus influenza* ⩾2 weeks before initiating treatment
Iptacopan (Fabhalta™)	December 6, 2023	Adult patients with PNH	Binds Factor B	Vaccinate against *N. meningitides, S. pneumonia* ⩾4 weeks before initiating treatment
Danicopan (Voydeya™)	March 29, 2024	Add-on therapy to ravulizumab or eculizumab in adult patients with PNH experiencing EVH	Binds reversibly to Factor D	Vaccinate against *N. meningitides, S. pneumonia* ⩾2 weeks before initiating treatment
Bkemv (Eculizumab-aagh)	May 28, 2024	Adult patients with PNH	Humanized mAb against complement C5, Eculizumab biosimilar	Vaccinate against *N. meningitidis*
Crovalimab (Piasky™)	June 24, 2024	Adult and pediatric patients ⩾13 years of age with PNH and body weight ⩾40 kg	mAb that binds C5	Vaccinate against *N. meningitidis* within 3 years before, or 7 days after initiating treatment
Epysqli (Eculizumab-aeeb)	July 22, 2024	Adult patients with PNH	Humanized mAb against complement C5, Eculizumab biosimilar	Vaccinate against *N. meningitides* within 3 years before initiating treatment

EVH, extravascular hemolysis; PNH, paroxysmal nocturnal hemoglobinuria.

#### Eculizumab biosimilars

There are currently two Eculizumab biosimilars on the US market. Bkemv (Eculizumab-aeeb) was the first approved by the FDA, in May 2024, and it is a monoclonal antibody that binds to complement C5 and inhibits activation of complement. Similar to Eculizumab, Bkemv carries a risk of serious meningococcal infections and thus vaccination against *Neisseria* spp. is advised. Bkemv was reported to have no clinically meaningful differences from Eculizumab and is thus considered an interchangeable biosimilar.^40^

Epysqli (eculizumab-aagh), formerly known as SB12, is the latest PNH medication approved by the FDA.^41^ It is another Eculizumab biosimilar which has shown equivalent pharmacokinetics including comparable binding to Fc gamma receptors and C1q.^42^ Patients receiving Epysqli had equivalent safety and immunogenicity profiles to those receiving Eculizumab in a phase III trial.^41^ In addition, these patients had similar levels of LDH after 6 months, representing similar breakdown of RBCs at this time point. Like Eculizumab and Bkemv, Epysqli also presents risk of meningococcal infection and thus vaccination is recommended.

#### Other agents on the rise

The latest research into PNH treatments have highlighted potential new targets, specifically, mannan-binding lectin-associated serine protease-3 (MASP-3) inhibitor. MASP-3 inhibitors such as OMS906 are under active research. These upstream inhibitors of complement are predicted to be more effective than anti-C5 therapies as they will prevent both intravascular hemolysis and EVH.^43,44^ Emerging therapies differ from those currently in clinical use by targeting multiple sites within the complement pathway. Notably, KP104, a bifunctional monoclonal antibody that inhibits C5 and activates factor H for proximal pathway regulation, is generating significant interest in the treatment of PNH. Interim results from a phase II study of KP104, a fusion protein targeting both terminal and alternative pathways, demonstrate significant clinical improvements in hemoglobin levels, LDH levels, and FACIT-fatigue scores, with no serious adverse events reported in complement inhibitor-naïve PNH patients.^45–47^

## Cost of PNH medications

PNH medications are extremely expensive. This means cost can be a major factor in patients’ care. These prices highlight the need to stay up to date on PNH medications to give patients the best results through the most cost-efficient method. Listed below is the estimated AWP pricing based on standard dosing for treatment in PNH (Table 2).

**Table 2.** Cost of FDA-approved PNH medications.

Medication	Treatment guidelines	REMS restriction	Price per unit	Estimated cost for 1 year
Eculizumab (Soliris™)	Induction: 600 mg IV weekly × 4 doses; maintenance: 900 mg IV on week 5, then 900 mg IV every 2 weeks	Ultomiris and Soliris REMS	300 mg/30 mL: AWP $7827.60	$626,208.00
Ravulizumab (Ultomiris™)	Intravenous dose based on weight (wt) • If wt 20 kg to <30 kg, loading dose: 900 mg IV × 1 dose; maintenance: 2100 mg IV every 8 weeks starting 2 weeks after the loading dose • If wt 30 kg to <40 kg, loading dose: 1200 mg IV × 1 dose; maintenance: 2700 mg IV every 8 weeks starting 2 weeks after the loading dose • If wt 40 kg to <60 kg, loading dose: 2400 mg IV × 1 dose; maintenance: 3000 mg IV every 8 weeks starting 2 weeks after the loading dose • If wt 60 kg to <100 kg, loading dose: 2700 mg IV × 1 dose; maintenance: 3300 mg IV every 8 weeks starting 2 weeks after the loading dose • If wt ⩾ 100 kg: loading dose: 3000 mg IV × 1 dose; maintenance: 3600 mg IV every 8 weeks starting 2 weeks after the loading dose Subcutaneous dose (SQ): wt ⩾ 40kg: weight based IV dosing then followed by 490 mg SQ weekly starting after IV loading dose	Ultomiris and Soliris REMS	300 mg/3 mL: AWP: $7684.80 1100 mg/11 mL: AWP: $28,177.60	For patients ⩾40 kg using IV only $599,414.40–$645,523.20
Pegcetacoplan (Empaveli™)	1080 mg SQ twice weekly	Empaveli REMS	1080 mg/20 mL: $5633.60	$585,894.40
Iptacopan (Fabhalta™)	200 mg by mouth twice daily	Fabhalta REMS	200 mg capsule (bottle of 60): AWP $54,246.60	$650,959.20
Danicopan (Voydeya™)	150 mg by mouth three times per day	Voydeya REMS	50 mg/100 mg carton pack (bottle of 180): AWP $4957.20	$59,486.40
Bkemv (Eculizumab-aeeb)	300 mg/30 mL (10 mg/mL) IV	Bkemv REMS	Price not yet set	Price not yet set
Crovalimab (Piasky™)	Loading dose • If wt ⩾ 40 kg to <100kg, 1000 mg IV over 60 min • If wt ⩾ 100 kg, 15,000 mg IV over 90 min SC injection once every 4 weeks • If wt ⩾ 40 kg to <100 kg, 680 mg IV • If wt ⩾ 100 kg, 1020 mg IV	Piasky REMS	Price not yet set	Price not yet set
Epysqli (Eculizumab-aagh)	Induction: 600 mg IV weekly, weeks 0–3; maintenance: 900 mg IV every 2 weeks	Epysqli REMS	Price not yet set	Price not yet set

AWS, average wholesale price; EVH, extravascular hemolysis; PNH, paroxysmal nocturnal hemoglobinuria; REMS, risk evaluation and mitigation strategy.

## Conclusion

Each of these medications represents a breakthrough in the treatment of PNH. From a disease that previously had no medication options to one with four and more on the rise, PNH remains an area of the active bench to bedside research. However, each medication has its own side effects, and physicians must be vigilant and recognize early the risk of EVH, BTH, and risk of infection with encapsulated organisms, necessitating vaccinations in patients before administering these medications.

## Declaration

**Ethics approval and consent to participate:** Not applicable.

**Consent for publication:** Not applicable.

**Availability of data and materials:** Not applicable.
